# Molecular identification of *Trichocera maculipennis,* an invasive fly species in the Maritime Antarctic

**DOI:** 10.1007/s11033-020-05566-5

**Published:** 2020-06-10

**Authors:** Marta Potocka, Ewa Krzemińska, Robert Gromadka, Jan Gawor, Joanna Kocot-Zalewska

**Affiliations:** 1 Department of Antarctic Biology, Institute of Biochemistry and Biophysics, Polish Academy of Sciences, Pawińskiego 5a, 02-106 Warsaw, Poland; 2 Institute of Systematics and Evolution of Animals, Polish Academy of Sciences, Sławkowska 17, 31-016 Kraków, Poland; 3 Laboratory of DNA Sequencing and Oligonucleotide Synthesis, Institute of Biochemistry and Biophysics, Polish Academy of Sciences, Pawińskiego 5a, 02-106 Warsaw, Poland; 4 Institute of Biology, University of Opole, Oleska 22, 45-052 Opole, Poland

**Keywords:** Invasive species, Antarctica, *Trichocera*, Non-native species, Molecular identification, Insects, Alien species

## Abstract

*Trichocera maculipennis*, an invasive Diptera, was described for the first time in Antarctica in 2006 in a sewage system of one of the scientific stations on King George Island, South Shetland Islands, and started to increase its distribution within the island. To date, only taxonomical description of this species, based on morphological data has been available, as there were no molecular data recorded. In the present study, we present two methods of molecular identification of this species—based on partial cytochrome c oxidase subunit I (COI) and 16S ribosomal RNA (16S) genes. An appropriate and easy-to-use assay for proper and fast identification of invasive species is a key requirement for further management decisions, especially in such a fragile environment as found in terrestrial Antarctica.

## Introduction

Terrestrial habitats in Antarctica are limited to ice–free areas, consisting 0.2% of the continent’s surface [1]. Most of these regions are located on the coast of the continent, especially the Antarctic Peninsula and associated archipelagos, and a number of oases in East Antarctica [2]. Severe conditions, such as extreme low temperature, limited primary production, and very strong wind, make Antarctica inhospitable for terrestrial organisms. Therefore, the region’s biodiversity is very low, with very simple ecological structure [3]. Flora is limited to cryptogams, such as lichens and bryophytes, and two native flowering species, *Deschampsia antarctica* and *Colobanthus quitensis*. Animal species are represented mostly by micro-invertebrates, and only two species of macro-arthropods, both chironomiid flies, *Parochlus steinenii* and *Belgica antarctica* [4–6]. However, the recent increase in human activity, together with climate change, observed in Maritime Antarctica in last 30 years [7], have led the region to become more exposed to expansion of non-native species, introduced unintentionally by national operators of scientific stations or tourists visiting the region. Most of these species are not adapted to survive in the Antarctic environment, but some have already shown good adaptation to harsh conditions and have now become invasive. Known examples are: a grass, *Poa annua* [8], a chironomiid midge *Eretmoptera murphyi* [3], and a tipulomorph fly, *Trichocera maculipennis* [9, 10]. Invasive species may cause serious threats for ecosystem structure and function [3].

*Trichocera (Saltrichocera) maculipennis*, described for the first time in Antarctica in 2006 in the sewage system of one of the scientific stations on King George Island, South Shetlands Islands [10], started to increase its distribution within the island [9]. To date, only taxonomical description of this species, based on morphological data, is available. There are few taxonomic specialists on the family and the genus *Trichocera*, and none are known to participate in Antarctic expeditions. For most introductions of non-native species to Antarctica it is difficult to obtain rapid specialist identification. This was identified as an important constraint in the expedited management of Antarctic invasions by Hughes and Convey [11] Therefore, a rapid and accurate method of identification to species level is sought in order to facilitate necessary and appropriate control and eradication measures. The aim of this report is to provide a simple but reliable approach to identification of this species based on molecular data. This uses two widely applied molecular barcoding techniques based on amplification and sequencing of COI and 16S mitochondrial rRNA (mtrRNA) gene fragments.

Several congeneric species were added to the analysis to enable species placement among the distant and close members of the genus, estimated as such on the basis of their morphological similarity. This is the first report of the two phylogenetically informative methods applied in *Trichoceridae* family.

*Trichocera maculipennis* Meigen, 1818, is a Holarctic species, known from Arctic to the southern regions of Mediterranean zone and Far East [12, 13]. In the Southern Hemisphere, this species has been described only from the Kerguelen Island [14], and recently from King George Island in Antarctica [9, 10]. The species was probably introduced to both southern locations by human agency [9, 15], and widely dispersed between scientific stations of King George Is. Recognition of potential introduction routes and the origin of the species is very important to prevent further invasions, and to enable the implementation of appropriate management strategies.

## Material and methods

### Material

Specimens of *Trichocera* (*Saltrichocera*) *maculipennis* Meigen, 1818: female [indicated as **Polar** in the diagrams], Arctowski Polish Station, data, collected from sewage system as described in [9] (IBB PAS, coll. B. Matuszczak); female **[CH]**: Switzerland, cave, 2. Galerie Sieben Hengste-Hohgant, alt. 1486 m, 26.12.1986–29.12. 1987 (MNHN, now ISEZ; coll. A. Hof); female: Poland, cave [PL] Pod Sokolą, 19.02.2018 (ISEA; coll. J. Zalewska). *Trichocera* (*Saltrichocera*) *regelationis* (L.), 1758, female, Poland, Ojców National Park, Wąwóz [gorge] Skałbania, 11.04. 1999 (ISEA, coll. A. Palaczyk); *Trichocera* (*Saltrichocera*) *nordica* Krzemińska & Gorzka, 2014 [16]: female, Finland, Oulanka Research Station, 5.09.2011; *Trichocera* (*Saltrichocera*) *parva* Meigen, 1804: female, Finland, Oulanka Research Station, 10.09.2011; *Trichocera* (*Trichocera*) *major* Meigen, 1818: female, Poland, Ojców National Park, Wąwóz [gorge] Korytania, 19.11.1992 (coll. E. Krzemińska). All specimens are housed in ISEA if not otherwise stated.

Institutional abbreviations.

IBB—Institute of Biochemistry and Biophysics, Polish Academy of Sciences, Warszawa, Poland.

ISEA—Institute of Systematics and Evolution of Animals, Polish Academy of Sciences, Kraków. Poland.

MNHN—Museé national d’histoire naturelle, Neuchâtel, Switzerland.

### Methods

#### DNA isolation from specimens

DNA isolation from insects was carried out as described by Gilbert et al. [17] with minor modifications as described below. The whole specimens were transferred into 1.5 ml microcentrifuge tubes and washed twice with 1 ml TE buffer (10 mM Tris–HCl, 1 mM EDTA pH 8.0) to remove residual ethanol from the sample, then 500 µl of lysis buffer (3 mM CaCl_2_, 2% (w/v) sodium dodecyl sulphate (SDS), 40 mM dithiotreitol (DTT), 50 µg/ml proteinase K, 100 mM Tris buffer pH 8 and 100 mM NaCl; final concentrations) were added. The homogenates were incubated overnight at 55 °C. Specimens were removed from the buffer, placed in 1 ml 95% (v/v) ethanol and replaced in their respective collections. The lysates were then extracted 3 times with an equal volume of phenol:chloroform:isoamyl alcohol (25:24:1; v/v/v) until the interface was clear. Nucleic acids were precipitated by addition of 0.7 volume of isopropanol. Three micro litres of glycogen (20 mg/ml) was added during the precipitation step to improve DNA yields. Samples were incubated at room temperature for 20 min and centrifuged using a MiniSpin Plus centrifuge (Eppendorf AG, Hamburg, Germany) at 14,000×*g* for 15 min. The supernatant was then removed and the DNA pellet washed twice in 500 µl room temperature 80% (v/v) ethanol, allowed to air-dry at 37 °C, and resuspended in 50 µl low-TE buffer (10 mM Tris pH 8.0, 0.1 mM EDTA). After isolation DNA quantity was measured using Qubit 3.0 fluorometer (Thermo Scientific, Waltham, USA) and High Sensitivity DNA quantification kit (Thermo Scientific, Waltham, USA). DNA concentration was normalized to final concentration of 5 ng/µl. All reagents used in the purification step were molecular biology grade and were purchased from Sigma.

#### PCR amplification and sequencing of mitochondrial barcodes

The standard cytochrome oxidase (COI) fragment was amplified using the primer pair described by Folmer et al. [18]:

LCO1490: 5′- GGTCAACAAATCATAAAGATATTGG-3

HCO2198: 5′- TAAACTTCAGGGTGACCAAAAAATCA-3'

16S mtrRNA fragment was amplified with following primer pair:

LR-N-13398: 5′-CGCCTGTTTAACAAAAACAT -3’

LR-J-12887: 5′-ACGCCGGTTTGAACTCAGATC-3’

described by Simon et al. [19].

PCR products were amplified using KAPA Robust PCR kit (Roche, Basel, Switzerland). PCR reactions were carried out in 20 µl final volume consisting of: 4 µl of KAPA 2G A buffer, 0.4 µl of 10 mM dNTPs, 1U of KAPA Robust polymerase (5 U/µl), 0.5 µl of each primer (10 µM), 11.45 µl of PCR-grade water and 2 µl of DNA template (10 ng). Amplification reaction conditions for both sets of barcoding primers were as follows: 3 min of initial denaturation at 95 °C, followed by 38 cycles of 30 s at 95 °C, 20 s at 50 °C, 30 s at 72 °C, and final extension period of 2 min at 72 °C. The amplified products were visualized through agarose gel electrophoresis (1.5%, wt/v) and ethidium bromide staining. The amplicons were purified using EPPiC Fast kit (A&A Biotechnology, Gdańsk, Poland) and directly sequenced with the same primers used for PCR amplification. Sanger sequencing was done using BigDye Terminator v3.1 chemistry and ABI3730xl genetic analyzer (Thermo Scientific, Waltham, USA).

#### Data analysis

Sequence data were analyzed using FinchTV ver. 1.4.0 (Geospiza, Akron, USA). Consensus sequences were obtained with Seqman Pro ver. 9.1 software (DNAStar, Madison, USA).

#### Molecular phylogenetic analysis by maximum likelihood method

##### Phylogenetic tree of mitochondrial 16S mtr RNA and and COI gene fragments

The evolutionary history was inferred by using the Maximum Likelihood method based on the Tamura-Nei model [20]. Evolutionary analyses were conducted in MEGA7 [21].

#### Nucleotide sequence submission and GenBank accession numbers

Accession numbers for each novel nucleotide sequence of COI and 16S mtrRNA genes of the *Trichocera* species are given in Table 1.

**Table 1:** List of species, collection sites, and GenBank accession numbers of COI and 16S mtrRNA sequences used in this study

Name	Collection site	COI accession number	16S mtrRNA accession number
*Trichocera brevicornis*		–	KC177458
*Trichocera maculipennis* CH	Switzerland, cave	–	MK356391
*Trichocera maculipennis* PL	Poland, cave	MK517414	MK356394
*Trichocera bimacula*		JN861750	JN861750
*Trichocera maculipennis* Polar	Polish Antarctic Station	MH378440	MK356395
*Trichocera major*	Poland, Ojców National Park	MK517411	MK356396
*Trichocera nordica* FIN	Finland, Oulanka National Park	–	MK356393
*Trichocera regelationis* PL	Poland, Ojców National Park	–	MK356392
*Trichocera parva*	Finland, Oulanka National Park	MK517412	MK356397
*Trichocera recondita*		MK517413	MK356398
*Trichocera regelationis*		MK517410	–
*Trichocera saltator*		GMGMF080-14.COI-5P [22]	–
*Drosophila melanogaster*		U37541	U37541

## Results

Eight specimens were subjected to a manual DNA isolation procedure. PCR amplification of 16S mitochondrial rRNA fragment was successful for all eight of the tested samples. In the case of COI fragment PCR was successful only for six samples. In the case of the two remaining specimens, they did not produce a visible amplification product. Several attempts were made to analyze additional molecular barcodes such as *nad6* or *cytb* using primer sets designed for Diptera [23] but only COI and 16S gene fragments were amplified successfully by PCR amplification. Obtained 16S mtrRNA and COI fragment consensus sequences were aligned against GenBank database using BLAST. The resulting sequence of the COI fragment was a 97% match to the *Trichocera* Barcode of Life Data system (BOLD) mitochondrial COI sequence (accession number KR386810.1) based on a BLAST comparison to the GenBank™ database. The 16S mtrRNA gene fragment showed 99% similarity to *Trichocera bimacula* mitochondrium sequence (accession number JN861750.1). Sequences obtained are listed in Table 1.

**Fig. 1:**
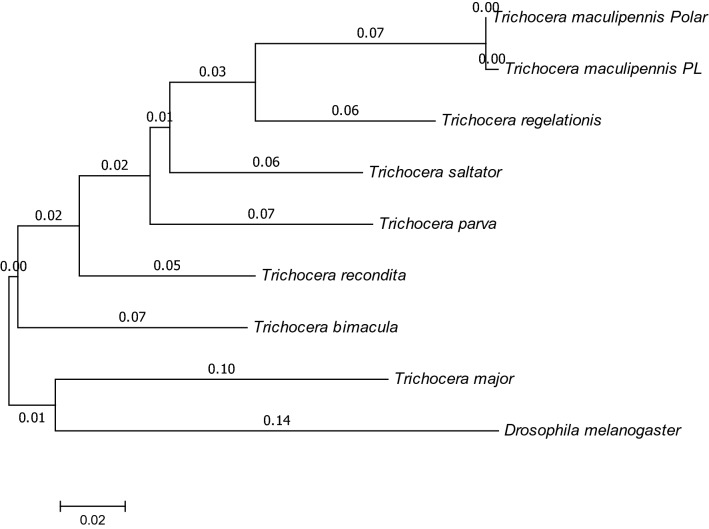
Evolutionary relationships of *Trichocera* species based on a fragment of mitochondrial COI gene

**Fig. 2:**
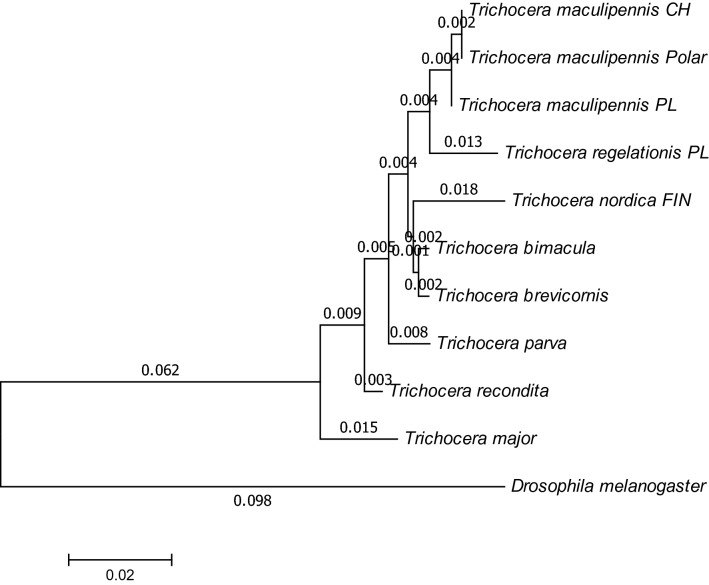
Evolutionary relationships of *Trichocera* species based on fragment of mitochondrial 16S ribosomal RNA gene

Molecular phylogenetic analysis by Maximum Likelihood method for both sequences are presented at Figs. 1 and 2.

Initial tree(s) for the heuristic search were obtained automatically by applying Neighbor-Join and BioNJ algorithms to a matrix of pairwise distances estimated using the Maximum Composite Likelihood (MCL) approach, and then selecting the topology with superior log likelihood value, using 1000 bootstrap. The highest log likelihood was (-2270,1219). The tree is drawn to scale, with branch lengths measured in the number of substitutions per site (next to the branches). The analysis involved nine nucleotide sequences. Codon positions included were 1st+2nd+3rd+Noncoding. All positions containing gaps and missing data were eliminated. There were a total of 526 positions in the final dataset.

Initial tree for the heuristic search were obtained automatically by applying Neighbor-Join and BioNJ algorithms to a matrix of pairwise distances estimated using the Maximum Composite Likelihood (MCL) approach, and then selecting the topology with superior log likelihood value, using 1000 bootstrap. The highest log likelihood was (-1218,9058). The tree is drawn to scale, with branch lengths measured in the number of substitutions per site (next to the branches). The analysis involved 11 nucleotide sequences. Codon positions included were 1st+2nd+3rd+Noncoding. All positions containing gaps and missing data were eliminated. There were a total of 513 positions in the final dataset.

## Discussion

Identification of *T. maculipennis* gave a satisfactory output by each method used (Figs. 1, 2); the specimens of this species from various localities, including those from the Polish Antarctic Arctowski Station (polar), cluster together. The sister branch is occupied by *Trichocera regelationis*, a closely related species, as inferred from similarity of morphological features (inner genitalia of male and female; spotted wing) based on which they were allotted previously to the *regelationis* group of species [24].

The remaining portions of both trees are difficult to compare, due to the separate sets of specimens and species analyzed in each.

The inner outgroup species is *Trichocera (Trichocera) major*, a representative of a different subgenus [25]; its sistergroup position to remaining trichoceriids is therefore expected and satisfactory. A branch next to the maculipennis + regelationis complex comprises two North American species, *T. (S.) bimacula* and *T. (S). brevicornis*, both similar to the species of the *regelationis* group; the former by spotted wings, the latter by male and female genitalia [26]. The position of *T. (S.) nordica* in the cluster is not so well supported by morphology of the genitalia.

Based on morphological data the method based on 16S ribosomal RNA gene would appear to be more consistent. *T. (S.) bimacula*, morphologically closely related to the maculipennis + regelationis complex, is located far on the COI-gene based tree.

Results presented herein could provide an important reference for future studies on *Trichocera* specimens, including new species identification projects and assessments of molecular variation between different geographical locations.

There were unfortunately some limitations to this study. The number of specimens collected for analysis was relatively small and our findings were potentially related to the limited number of specimens in collections. More extensive investigations with a larger number of samples are required for the future studies and for definitive findings to be made.

The presented data is a response to the Committee for Environmental Protection (CEP) recommendations regarding development of a standardized monitoring program to effectively control the spread of the flies in Maritime Antarctica, and identify a practical and coordinated management response for fly eradication [27, 28]. In this regard, use of molecular tools for proper and rapid identification of invasive species are key to improved management decisions, especially in such fragile environment as terrestrial Antarctica.
